# SHERPA-city: A web application to assess the impact of traffic measures on NO_2_ pollution in cities

**DOI:** 10.1016/j.envsoft.2020.104904

**Published:** 2021-01

**Authors:** B. Degraeuwe, E. Pisoni, P. Christidis, A. Christodoulou, P. Thunis

**Affiliations:** a European Commission, Joint Research Centre (JRC), Ispra, Italy; b European Commission, Joint Research Centre (JRC), Seville, Spain

**Keywords:** NO_2_, Traffic, Low emission zone, LEZ

## Abstract

This paper presents SHERPA-City, a web application to assess the potential of traffic measures to abate NO_2_ air pollution in cities. The application is developed by the Joint Research Centre. It is freely available (https://integrated-assessment.jrc.ec.europa.eu) and allows the user to perform a fast screening of possible NO_2_ abatement measures addressing traffic in European cities. SHERPA-City results depend on the quality of the default input data. It is therefore important to stress that the SHERPA-City default traffic flows, emission factors, fleet composition, road network topology, NO_2_ pollution from other sources and meteorological data are based on EU-wide datasets that may not always represent perfectly a particular local situation. This is why the SHERPA-City allows the default data to be substituted by local data, to better reflect local features. This tool must be considered as a first step in exploring options to abate NO_2_ air pollution through transport measures. The final decisions should be based, wherever possible, on full-scale modelling studies incorporating local knowledge.

## Highlights

• A free user-friendly web application to evaluate the impact of traffic measures on NO_2_ concentrations in European cities.
• EU wide default traffic data and emission factors are provided.
• Possibility to upload own traffic data and emission factors.
• A case study on Madrid Low Emission Zones demonstrates the key features of the tool.

## Graphical abstract

**Figure:**
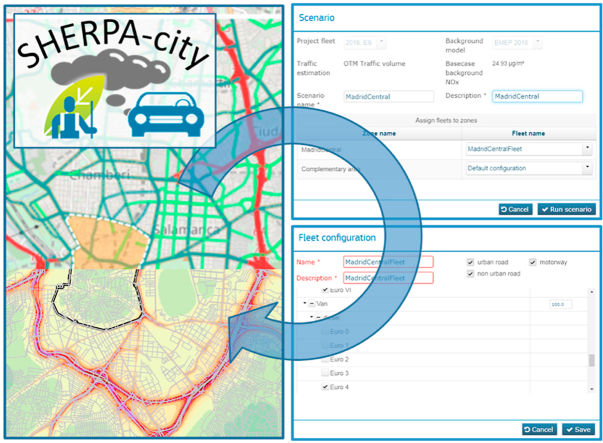

## 1 Introduction

Many cities in Europe still suffer from poor air quality. One of the major causes, besides particulate matter (PM), is nitrogen dioxide (NO_2_). It has a direct negative impact on health and an indirect one through the formation of fine secondary particulate matter (PM2.5) and ozone (O_3_) (WHO-Europe, 2013). Though the situation has improved over the last 30 years, air pollution remains the single largest environmental health risk in Europe according to the World Health Organization (WHO, 2018). Both the Ambient Air Quality Directives (EC, 2008) and the WHO Air Quality Guidelines (AQG, WHO 2005) prescribe a limit value of 40 μg/m^3^ for NO_2_ as an annual average ambient concentration. In their latest Air Quality in Europe report (EEA, 2019), the European Environmental Agency indicates that many European cities still exceed this limit value for both NO_2_ and PM2.5. In 2017, around 10% of all EU28 air quality monitoring stations reported exceedances of this limit value (EEA, 2019; Thunis et al., 2018) with about 86% of them occurring at traffic stations, i.e. stations placed close to major roads. This is to be expected, as traffic is a major source of NO_X_, which forms NO_2_ when reacting with O_3_ in the atmosphere.

To respond to this air pollution challenge, cities often take measures to reduce air pollution in areas with high traffic densities (see Pisoni et al. (2019), for a review of such measures). Such measures are sometimes required to comply with the AAQD, and include access restrictions to limit transport emissions in cities. The EU Commission's (EC, 2014) study on a European City Pass for Low Emission Zones (LEZs) showed that there is a patchwork of LEZ approaches and rules applied throughout the EU. The effectiveness of LEZs depends strongly on the way they are implemented; which emission standards are allowed, for which vehicle categories restrictions are in place, the type of access control and particularly the number of exemptions granted. Studies like Font et al. (2019) proof that vehicle emission regulation combined with other traffic policies can have a positive impact on air quality in Paris, London and other cities. The authors analysed trends in PM and NO_2_. They found that the introduction of Euro V emission limits for trucks combined with a LEZ had a positive impact in London. However, they also detected that more stringent NO_X_ emission limits for Euro 5 diesel cars had no positive impact because these cars performed worse on the road, a consequence of high emission factors in real driving conditions.

To correctly estimate the impact of a LEZ and other traffic measures, modelling tools are helpful. Many tools are available (Jensen et al., 2017; Oettl and Uhrner, 2011; Lefebvre et al., 2013; Yeganeh et al., 2018; Rahman et al., 2017; Sokhi et al., 2008) to model yearly, daily and hourly particulate matter, and nitrogen dioxide concentrations at street level, taking into account urban topography, emission performance of vehicles, the composition of the vehicle fleet, the daily activity patterns, and background pollution. These tools can be used to perform ex ante evaluations, to understand the impact of a LEZ on air quality. However, they require appropriate IT infrastructure and the availability of detailed input data. As a result, in many cases, municipalities wishing to put into place access restrictions do not have access to a simple tool to estimate the effects of such measures on air quality before they are applied. Thus, they have no way to evaluate access restrictions, neither geographically nor as a function of emission performance. To overcome this limitation, the Joint Research Centre (JRC) developed the SHERPA-City tool. This simplified screening tool mimics a Gaussian pollutant dispersion model, but with a much shorter calculation time. It can therefore be used to evaluate the impact of traffic management measures leading to reductions of emissions at the source.

## 2 Methods

The methodology behind the SHERPA-city web application, consists of the following steps:

1. Selection of a modelling domain of maximum 100 km^2^ within one of the EU countries (EU27 + CH + UK + NO). The application provides the full road network for the specific domain, as well as the corresponding traffic levels (Annual Average Daily Traffic, AADT) available in the database.
2. Implementation of desired infrastructure changes (e.g. add or close roads, different traffic flows) and definition of zones where specific traffic policy will apply.
3. Selection of the vehicle fleet. Default emission factors per country, year and road type (both historical and forecasts) are available to support this choice.
4. Definition of traffic measures that modify the fleet composition and/or traffic flows. Road specific emission factors are calculated per road type and per domain zones.
5. Combination of the road network and emission factors to generate gridded traffic emissions at high resolution.
6. Application of atmospheric ‘dispersion kernels' (Masey et al., 2018) to convert traffic emissions into contributions to the annual average concentration. This kernel is location dependent.
7. Combination of the local traffic contribution with contributions from other emission sources within the domain as well as from all sources outside the domain.
8. Conversion from NO_X_ to NO_2_ concentrations.

In the following paragraphs these steps (and how they are implemented in SHERPA-city) are explained in detail.

### 2.1 Road network and traffic data

The SHERPA-City application uses traffic data from OpenTransportMap (OTM) (Jedlička et al., 2015, [Bibr bib10][Bibr bib11][Bibr bib10]) and network information from OpenStreetMap (OSM) (OpenStreetMap contributors, 2020). In particular, results from the OMNITRANS model^1^ are used. These result are represented by a road network with these attributes: ‘traffic volume’ and ‘capacity’, that are expressed using the concept of ‘annual average daily traffic’ (AADT). It is however not possible to use this dataset as such because the sum of the annual vehicle kilometres per country does not match the national totals, neither for traffic volume nor for capacity. The annual vehicle kilometers per country of OTM are on average 37% (SD = 24%) different (smaller) than the official ones reported i.e. by GAINS (IIASA, 2020). The traffic volume based on road capacity on the contrary in general overestimates the GAINS data by 4.5 times (SD = 3%) because in reality roads are not used all the time at full capacity. Hence we decided to scale the OTM data to the GAINS national total annual vehicle kilometres. In the case of traffic volume, some gap filling is necessary. For example, no traffic is allocated by OTM on OSM fourth class roads (small urban roads) or in a few NUTS3 regions, traffic is missing on other roads. To generate traffic on these roads, a correlation between the functional road class and the traffic volume is used. There is indeed a downward linear trend between the road class and the logarithm of the OTM traffic volume. This means that the traffic volume decreases with a constant percentage from one class to the next. The average decrease across all countries is 64% (from one class to another). This correlation is extrapolated to the fourth class roads for each country. In a next step the AADT on all roads is scaled up with the same factor to match the GAINS national totals. This road network is available as the default in SHERPA-city. It is also possible to choose the scaled road capacity if the user judges that this dataset is closer to reality. If neither of the two dataset is good enough the user can edit the existing dataset or upload his own data. A comparison between the default traffic dataset and local data is presented in the case study for Madrid, shown in the next sections of this manuscript.

### 2.2 Road transport emissions

Besides the traffic flow, the fleet composition and emission factors are also needed to calculate emissions on each road segment. The fleet composition is required in terms of kilometers driven for each segment of the fleet, i.e. vehicle categories (passenger cars, vans, trucks, busses, mopeds and motorcycles) and sub-segments of these categories (size, fuel and emission standard). The fleet composition for SHERPA-city is acquired from EMISIA (https://www.emisia.com/). Vehicle numbers, kilometers driven and corresponding emissions are provided according to the COPERT methodology (Ntziachristos et al., 2018). However, SHERPA-city uses an aggregated version of the COPERT classification of the vehicle fleet with only 77 categories instead of 325. This makes it easier to configure a fleet and still allows for modelling typical traffic restrictions. The user can control the following fleet categories:

• Passenger cars. The technologies considered are gasoline, diesel, LPG, CNG, hybrid and electricity. All Euro-norms are considered: pre-Euro, Euro 1 to 6 and Euro 6c and Euro 6 d (for diesel cars). The data are aggregated at vehicle size.
• Vans. Only diesel and gasoline are considered for all Euro norms, up to 6c.
• Trucks. Only diesel is considered with distinction between Euro-norms, from pre-Euro to Euro VI.
• Busses. Diesel and CNG are considered with distinction between Euro norms (from pre-Euro to Euro VI)
• Motorcycles. This is one single category, without distinction between sizes or norms.
• Mopeds. This is one single category, without distinction between sizes or norms.

**Fig. 1:**
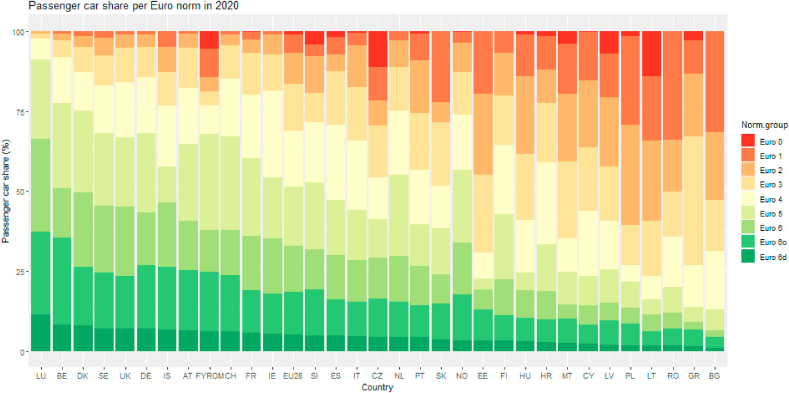
Share of each Euro norm for passenger cars per country (own elaboration from COPERT data).

EMISIA also provides the share of vehicle kilometers of each vehicle category per road type. E.g. the share of trucks is bigger on highways than on urban roads. With this information the emission factors of specific vehicle classes can be weighted by their respective kilometers driven to obtain a fleet emission factor per road type. For the past years until 2016 the numbers are based on national statistics while for future years (2020 and 2025) predictions are made with the EMISIA's Sybil software. This model predicts fleet composition starting from the last data year. It takes into account scrappage, second hand imports and sales of new cars. Fig. 1 shows the share of passenger cars complying with each Euro norm per country in 2020.

The SHERPA-city application assumes that the local fleet mix corresponds to the national one. This is however not always the case as differences between urban and rural fleets or between fleets in different regions within the same country can be substantial. If better estimates are available, they can be uploaded by the user. An example file is available in the fleet configuration module of the web application.

The SHERPA-city web application has a fleet configuration module that allows to reduce the mileage or completely ban a part of the fleet. When a subcategory is banned (e.g. passenger cars using diesel fuel, or passenger cars compliant with Euro 3 or older) the vehicle kilometers of the remaining categories are scaled up to the kilometers of that respective category. It is assumed that the activity of a banned subcategory will be replaced by the remaining, allowed vehicles in that category. If the user wants to model a reduction of the kilometers driven in a category, on top of a fleet composition change, this must be introduced separately. On the other hand, if a whole category is banned, its vehicle kilometers are not moved to other categories. This means that removing all trucks will not increase the kilometres driven by busses, cars, etc.

The final step in creating emissions consists in converting the road network into a raster. The intersection between grid cells and roads determines the level of emissions in each cell. The network is finally converted in the local UTM projection at a spatial resolution of 20 m.

### 2.3 Dispersion kernels

**Fig. 2:**
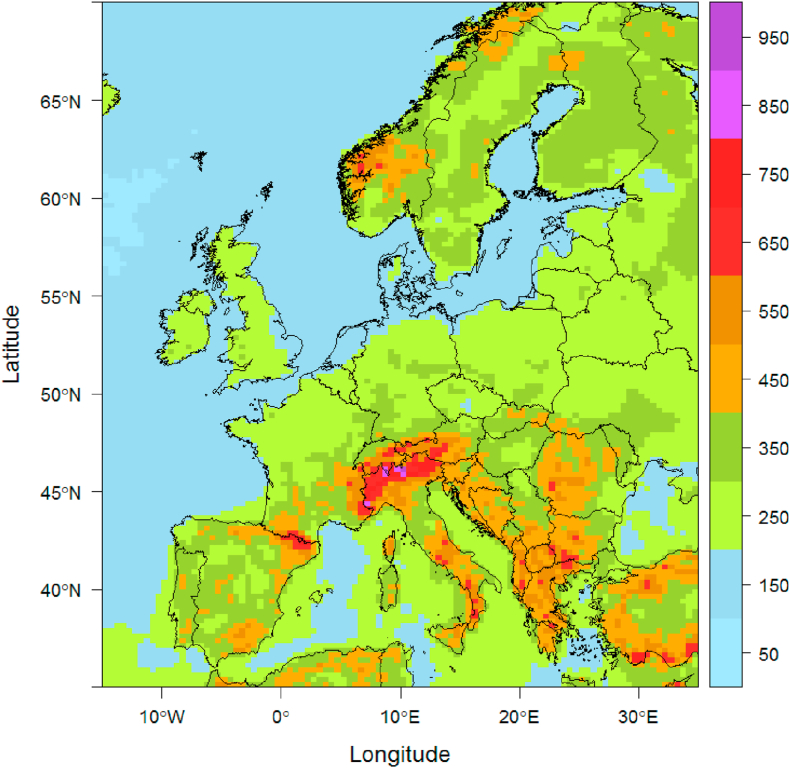
Maximum annual average concentration (*μ*g/m^3^) around a point source of 1 kg/h, resolution 28 × 28km, 14,000 kernels (own elaboration).

To calculate annual average concentrations, dispersion kernels are used. A dispersion kernel provides information on the average annual concentration of a pollutant (NO_X_ or PM2.5) around a normalized emission source (1 kg/h was chosen). Kernels covering Europe are pre-calculated over a 0.2^∘^ latitude and 0.5^∘^ longitude resolution grid between 35^∘^ and 70^∘^ North and between 15^∘^ West and 35^∘^ East, resulting in 14,000 kernels. For a project designed in a particular city the closest kernel is then chosen. Kernels are calculated with the Gaussian dispersion model IFDM (Lefebvre et al., 2013). With hourly wind speed and temperature data as input, concentrations around a unit source of 1 kg/h within a domain of 4 by 4 km are calculated at a resolution of 20 by 20 m. The IFDM hourly output concentrations are then averaged over the year. The shape of a dispersion kernel depends on the local meteorology. At locations characterized by higher wind speeds, polluted air is dispersed more efficiently and concentrations are therefore lower. Fig. 2 shows the maximum concentration obtained for each kernel. High values for the kernel are found in mountain areas while lower concentrations are found at sea or in coastal locations (where higher wind speeds spread out air pollution more efficiently).

To calculate the traffic contribution to the concentration for each cell, the source kernel is multiplied by the strength of the emissions in that cell. These scaled kernels are then summed over the entire domain.

### 2.4 NO_X_ emissions from other sources: the background

To obtain the total concentrations, background levels must be considered. We use a simulation from a chemistry transport model (CTM) to obtain this information. The following options are available:

• An EMEP (Simpson et al., 2012) run for the year 2015. This run gives a concentration value for every cell within the domain at a resolution of 0.1 by 0.1°. This corresponds roughly to 11 km latitude and 7 km longitude.
• A CHIMERE (Mailler et al., 2017) run for the year 2010. This run gives a concentration value for every cell within the domain at a resolution of 0.0625 by 0.125°. This corresponds roughly to 7 km latitude and 7 km longitude.

Regardless of the selected option, the CTM provides one value for NO and NO_2_ that is interpolated to the centre of the city SHERPA domain. This concentration also includes the contribution of local traffic. To avoid double-counting the average concentration due to local sources (the result of the kernel approach) is subtracted from the CTM value. Then the high resolution local contribution is added to this corrected CTM concentration. In the case of NO_2_, this correction is carried out on the NO_X_ concentrations. In a final step, explained in the next paragraph, the NO_2_ concentration is calculated from the NO_X_ concentration.

### 2.5 From annual average NO_X_ to NO_2_

SHERPA-City does not calculate the annual average NO_2_ directly. First the annual average NO_X_ concentration is calculated as a combination of a corrected CTM background and the local contribution (see previous section). Then the NO_2_ concentration is calculated using a correlation between the NO_2_ fraction and the total NO_X_ concentration, as a function of the overall NO_X_ emissions. This approximation is justified because close to the source and shortly after the emission NO_X_ behaves as an inert gas. Therefore its dispersion is well predicted with a Gaussian dispersion model and the local contribution to the concentration is proportional to the NO_X_ emissions. The reactions that remove NO_X_ forming nitrates and secondary PM are much slower and accounted for by the CTM. The only important reactions close to the emissions release point are the reaction between ozone and nitrogen monoxide and the photolysis of nitrogen dioxide. These reactions do not change the total amount of NO_X_.

(1) $$NO_{2}+O_{2}+h\nu \Leftrightarrow NO+O_{3}$$

When solar radiation shifts the equilibrium to the ‘right’ of the aforementioned equation, ozone is formed. In the absence of solar radiation the equilibrium shifts to the left and NO_2_ is formed. The resulting annual average NO_2_ fraction therefore depends on local conditions like the concentration of ozone and other oxidants, solar radiation and the presence of volatile organic carbons (VOC). The trend is similar everywhere. When NO_X_ concentrations are low, the NO_2_ fraction increases to 100%; when NO_X_ concentrations are high the NO_2_ fraction levels off. In this ozone limited regime not all the NO can be oxidized to NO_2_. This means that a reduction of NO_X_ emissions has a smaller impact on NO_2_ concentrations when the NO_X_ concentrations are high. So, this effect is of interest when planning measures to reduce pollution. Romberg et al. (1996) and Bächlin et al. (2008) proposed an empirical correlation approximating this behaviour with a formula like equation (2).

(2) $$f_{NO_{2}}=\frac{{\left[NO_{2}\right]}_{a}}{{\left[NO_{X}\right]}_{a}}=\frac{a}{{\left[NO_{X}\right]}_{a}+b}+c$$

${\left[NO_{X}\right]}_{a}$ and ${\left[NO_{2}\right]}_{a}$ are the annual average NO_X_ and NO_2_, respectively. Romberg et al. (1996) proposed *a* = 103, *b* = 130 and *c* = 0.005. Bächlin et al. (2008) proposed *a* = 29, *b* = 35 and *c* = 0.217. Jurado et al. (2020) compared both formulas with measurements in France and found that both models perform similarly, with respective deviations of 9.1% and 10.2%. A third model by Derwent and Middleton (1996) was shown to perform slightly better but requires hourly data and cannot be used for this application.

**Fig. 3:**
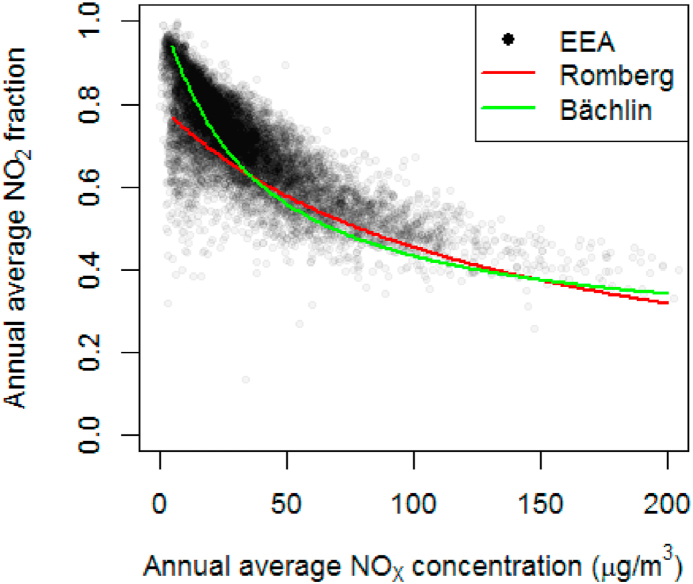
Measured NO_2_-fraction versus NO_X_ (annual average) and the empirical Romberg and Bächlin correlations.

Fig. 3 shows the Romberg and Bächlin correlations applied to all annual average data available in EEA Air quality statistics (EEA, 2020). The correlations follows the trend but are not accurate. Therefor SHERPA-city uses a correlation with adjusted coefficients so that the background CTM value in the domain lies on the curve. This adapted correlation will be used to calculate the NO_2_ concentration due to changing NO_X_ emissions.

## 3 The web application

The SHERPA-city web application implements all the aforementioned steps. It is accessible through a login page: https://integrated-assessment.jrc.ec.europa.eu/sherpacity. The use of SHERPA-city is free, and registration can be done by selecting “register” on the login page or directly through the page: https://aqm.jrc.ec.europa.eu/register.aspx. Users will receive an e-mail by the site administrator granting access to the application.

The steps to set up a simulation in the SHERPA-city web application accessible are:

• Creating a new project and selecting national average default fleet compositions from previous or for future years. Fleets for all EU27 countries, UK, Switzerland and Norway are available.
• Selecting the domain (study area) for the simulations. The maximum size is 100 km^2^.
• Creating zones or modifying network and traffic data. This step can be carried out through the web application or by uploading user data. This step allows the user to create the zones where traffic measures, defined in the fleet configurations (see below), can be applied. It also allows the user to modify the default traffic data (e.g. by modifying traffic flow for specific roads in the domain) and network (e.g. by removing or adding streets). This can be done through the web application or by uploading the user's own shape files.
• Creating new fleet configurations that modify the default fleet in terms of a) traffic volumes by vehicle type; b) allowed vehicles by type, fuel or Euro standard; c) type of road where the measures are applied. Fleets can be defined at any moment of the workflow before the simulation of a scenario, and applied in any project. In combination with the default project fleet, a fleet configuration becomes then a real fleet. E.g. a user can create a fleet configuration without trucks. This configuration can be combined in a project with i.e. the Bulgarian fleet of 2016 or the Portuguese one of 2025.
• Simulating the basecase and other scenarios is the core of the Sherpa-city web application. After the simulation of the basecase, the user can define scenarios to compare results. The scenarios are defined, within a project, by selecting a specific fleet configuration to be applied to each zone.
• Visualizing results of the simulation is the final step. Both concentrations and emissions can be visualized as gridded values, average values and in table or chart format. Results can be exported in raster format.

Each step is described in more detail in the manual, that can be found on the tool website. The import of shape files and other known issues are reported in the Annex of the manual. During the workflow, the user can visualize and update the process status by clicking on the “verify process status” and “update process status” buttons at the top-right of the page.

Also an offline version of the tool is available on github (at https://github.com/bd77/SHERPA-city). This offline version is also used to implement the case study, presented in the next section.

## 4 Case study: Madrid Central

To illustrate how the SHERPA-city web tool works, an application has been implemented to study the ‘Madrid Central’ case. Madrid introduced a low emission zone, called *Madrid Central*, on November 30, 2018, in which only specific vehicles could enter. A classification of vehicles according to their environmental impact was introduced by the transport ministry:

• zero emissions vehicles: mainly electrical vehicles.
• ECO vehicles: hybrid, natural gas, LPG vehicles.
• type C vehicles: gasoline passenger cars and vans complying with Euro 4/IV, 5/V o 6/VI, diesel cars, vans and trucks complying with EURO 6/VI.
• type B vehicles: gasoline passenger cars and vans complying with Euro 3/III, diesel cars, vans and trucks complying with Euro 4/IV and 5/V.
• type A vehicles: all vehicles not contained in the previous classes.

Vehicles are identified with a sticker. In the LEZ vehicles of type A are not allowed. These are mainly gasoline vehicles build before the year 2000 and diesel cars older than 2005. Vehicles of class B and C can access to park. ECO and zero emission vehicles can circulate without restrictions. More details are available on the website of the city of Madrid (Ayuntamiento de Madrid, 2020). In this paragraph first the available input data are described, and then few scenarios are run to evaluate the impact of the LEZ on NO_2_ concentrations.

### 4.1 Traffic and fleet data

**Fig. 4:**
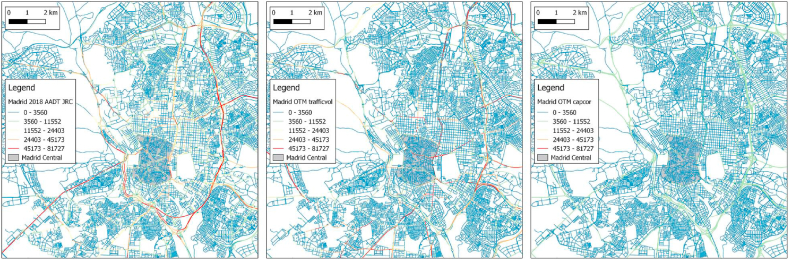
Comparison of road traffic data for Madrid: local data (left), OTM trafficvol (middle) and OTM capacity (right).

Traffic counts are available for 2018 and 2019 at about 2000 locations in Madrid. These were used to calibrate a traffic model providing traffic flows on all roads in the Madrid area. These local data are compared with the SHERPA-city default dataset. Table 1 shows the vehicle kilometers driven (AADT, that is to say Annual Average Daily Traffic) in the metropolitan area split in two areas; the Madrid Central LEZ and the rest of the area outside the LEZ. According to the traffic data of Madrid the traffic in the LEZ represents only 1.2% of the traffic in the metropolitan area. The default datasets provided in SHERPA city give 1.5% and 0.8% for the OTM traffic volume and OTM road capacity, respectively. Fig. 4 shows maps of the three datasets on the same scale. The map on the left are the data provided by Madrid. The map in the middle are the OTM trafficvol data. There are clear differences, especially on the M30 highway surrounding the city and on major roads in the centre. The OTM data underestimate the traffic on some parts of the highway and overestimate the traffic on some major roads in the centre. The OTM capacity dataset smears out the traffic evenly all over the domain. In the next section the local Madrid data will be used, because of their higher quality in comparison to the default data.

**Table 1:** AADT in the Madrid metropolitan area and the LEZ according to local data and the default datasets of SHERPA-city in Mvkm and percentage of the total kilometers.

Zone	Madrid 2018	Madrid 2019	OTM trafficvol	OTM capacity
Outside LEZ	20.97 (98.8%)	20.79 (97.9%)	30.2 (98.5%)	14.73 (99.2%)
Madrid Central	0.26 (1.2%)	0.25 (1.2%)	0.47 (1.5%)	0.11 (0.8%)

### 4.2 Scenarios

The aim of this SHERPA-city application is to understand the impact of the Madrid LEZ on NO_2_ concentrations. To disentangle the impact from the LEZ and traffic changes the following scenarios were calculated, considering:

• 2018 local traffic data, allowing all the fleet to travel everywhere,
• 2018 local traffic data, with only authorized fleet in the *Madrid Central* LEZ,
• 2019 local traffic data, allowing all the fleet to travel everywhere,
• 2019 local traffic data, with only authorized fleet in the *Madrid Central* LEZ.

In this way it is possible to differentiate the impact caused by traffic changes (independent from the LEZ) from the impact caused by the LEZ implementation itself. An additional test is done, in which we completely remove traffic from the LEZ, to see which could be the most extreme air quality improvement (i.e. keeping fixed the area of reduction but reducing to zero the allowed fleet).

### 4.3 Concentration results

**Fig. 5:**
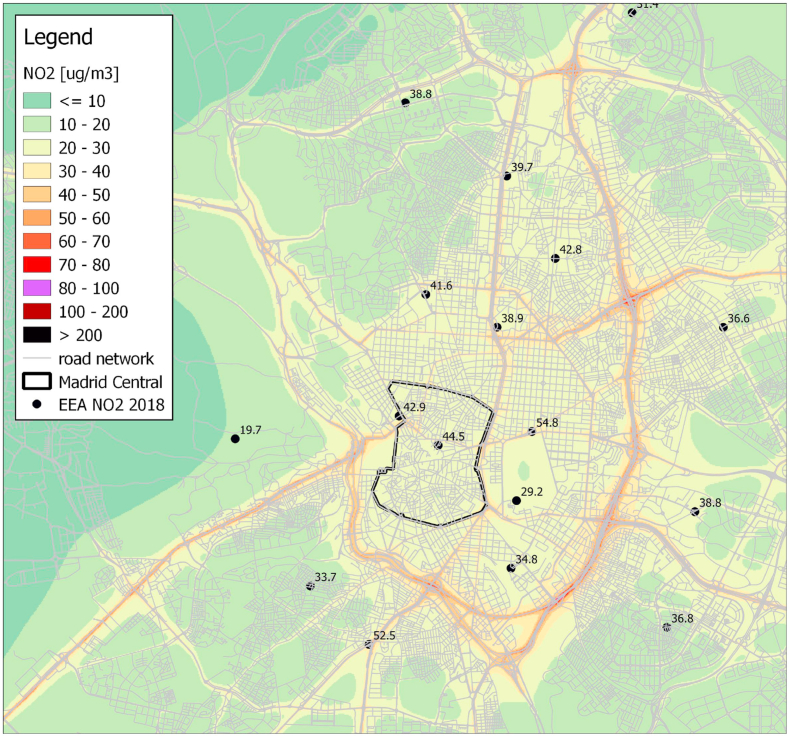
NO_2_ concentration in Madrid in 2018 without LEZ (this map was produced with QGIS, using the network and concentrations exported from SHERPA-city web application).

In this section we show the results of the SHERPA-City application. It is important to stress that results are shown both for NO_X_ (output of the SHERPA-City) and NO_2_ (as obtained applying the NO_X_ to NO conversion described in previous sections). As an example, Fig. 5 shows the NO_2_ concentration in Madrid in 2018 as predicted by SHERPA-city. The maps shows how SHERPA-City capture the spatial trends of NO_2_ (higher on main roads, lower when distant from roads) with a reasonable agreement with observations (see points and values on the map).

Table 2 shows the average (over the LEZ) results obtained for NO_X_ and NO_2_. In particular showing the basecase, the case regulated by the LEZ (banning the A-type vehicles) and the extreme case with no traffic on the LEZ. Looking at both years 2018 and 2019 (in Table 2), it is clear how the NO_X_ and NO_2_ reductions are quite limited for the mean values, going from the basecase to the LEZ scenario. Only a more aggressive LEZ (going to a very small allowed traffic in the LEZ) would reduce i.e. NO_2_ (in 2019) from 23.9 to 19.9 μg/m^3^ (while LEZ simulated case stops at 23.6 *μ*g/m^3^). This small impact is due to the small amount of AADT reduced with the LEZ (Table 1). Looking at the maximum values (instead of the mean values, again over the LEZ), the impact is higher, i.e. moving (for 2019) from 54.5 (basecase) to 41.4 (no traffic) *μ*g/m^3^. However, one should remember that LEZ are done usually not only for air quality, but more in general to improve quality of life (reducting noise, accidents, …, allowing for more walking, biking, …); so the results of this analysis with SHERPA-City is a very partial view of the issue at stake (representing only one of the indicators to be considered). In addition to this, the LEZ discussed here is designed in such a way that only a very small fraction of the city transport is affected. Stronger impacts would require more ambitious LEZ configurations, particularly in terms of spatial extension.

**Table 2:** Average and maximum NO_X_ and NO_2_ concentration in the *Madrid Central* LEZ in 2018 and 2019 for three scenarios: no change, LEZ and no-traffic.

Scenario	Traffic year	Total NO_X_ (*μg*/*m*^3^)	Traffic NO_X_ (*μg*/*m*^3^)	NO_2_ (*μg*/*m*^3^)
		Mean/Max	Mean/Max	Mean/Max
Basecase	2018	35.0/119.3	23.6/108.2	24.1/53.9
No A-class vehicles	2018	34.3/116.2	22.9/105.2	23.7/53.1
No traffic	2018	27.1/77.8	15.7/66.8	19.9/41.6
Basecase	2019	34.7/120.9	23.3/109.9	23.9/54.4
No A-class vehicles	2019	34.0/117.7	22.7/106.7	23.6/53.5
No traffic	2019	27.0/77.2	15.6/66.2	19.9/41.4

## 5 Discussion and conclusions

In this paper we have presented a novel user-friendly web application (SHERPA-city), to assess traffic measures in cities. In particular, SHERPA-city can be used to evaluate the impact of Low Emission Zones (LEZ) on NO_2_ and PM concentrations. In comparison to other existing approaches, it can be used with limited effort (using default data) to test the impact of mobility measures on air quality. However, it is advisable to replace the SHERPA-city default data with local data, that likely reflect better the local traffic and fleet composition; or at least, to check for a given study domain, if the default data respect real local conditions. Also a case study on Madrid has been presented, with a focus on the evaluation of a Low Emission Zone implemented few years ago. Results, using locally provided data, show that the LEZ, affecting only a small portion of the total AADT of Madrid, had in reality a small impact on NO_2_ average concentrations (and a bit higher for maximum values). Summarizing, we think SHERPA-city can be used as a screening tool to check ex-ante or ex-post the impact of LEZ on pollutant levels at a very fine spatial resolution. The main advantages of using SHERPA-city are:

• the availability of road networks with default traffic data, fleet data and emission factors, covering the whole EU;
• the possibility to select domains and zones for traffic policies at a fine spatial resolution over a detailed map;
• the possibility to define traffic measures in terms of fleet composition and traffic volumes;
• the calculation of contributions from other sources than traffic, from a full air quality model;
• the possibility to compute scenario concentrations, to be compared with a baseline scenario.

Applying the default values in SHERPA-city can give an approximation of the real impacts, for the various EU city. So, introducing local data on traffic, fleet mix and background concentrations is recommended in order to achieve a better correspondence of the model results with real local conditions.

## Software availability

The SHERPA-city tool can be accessed at: https://integrated-assessment.jrc.ec.europa.eu (after registration, free of charge). The source code for the case study shown in this paper is available at: https://github.com/bd77/SHERPA-city.

## Disclaimer

The views expressed in this paper are purely those of the authors and may not, under any circumstances, be regarded as an official position of the European Commission.

## Declaration of competing interest

The authors declare that they have no known competing financial interests or personal relationships that could have appeared to influence the work reported in this paper.

## Footnotes

1 https://www.dat.nl/en/solutions/transport-modelling-omnitrans/.
